# Efficacy of the Mediterranean Diet Containing Different Macronutrients on Non-Alcoholic Fatty Liver Disease

**DOI:** 10.3390/nu16162699

**Published:** 2024-08-14

**Authors:** Vahibe Uluçay Kestane, Murat Baş

**Affiliations:** 1 Department of Nutrition and Dietetics, Institute of Health Sciences, Acibadem Mehmet Ali Aydinlar University, Istanbul 34752, Turkey; 2 Department of Nutrition and Dietetics, Institute of Health Sciences, İstanbul Galata University, Istanbul 34432, Turkey; 3 Department of Nutrition and Dietetics, Faculty of Health Sciences, Acibadem Mehmet Ali Aydinlar University, Istanbul 34752, Turkey

**Keywords:** non-alcoholic fatty liver disease, steatohepatitis, Mediterranean diet, liver enzymes, fatty liver index

## Abstract

This study aimed to investigate the effects of the typical Mediterranean diet (TMD), low-carbohydrate Mediterranean diet (LCMD), and low-fat Mediterranean diet (LFMD) on biochemical findings, fatty liver index (FLI), anthropometric measurements, and body composition in individuals with obesity with non-alcoholic fatty liver disease (NAFLD) and insulin resistance. This study included 63 participants with obesity with insulin resistance diagnosed with NAFLD by ultrasonography to investigate the effects of an 8-week energy-restricted TMD, LCMD, and LFMD on biochemical findings, FLI, fibrosis-4 index (FIB-4), anthropometric measurements, and body composition. Patients were randomized into three groups and were interviewed face-to-face every week. According to the food consumption records (baseline end), the difference in the amount of sucrose and total fat consumed in the TMD group; the difference in energy intake from sucrose, monounsaturated fatty acids, and oleic acid in the LCMD group; and the difference in energy intake from fiber, sucrose, monounsaturated and polyunsaturated fatty acids, and cholesterol in the LFMD group showed significant correlations with liver enzymes and FLI (*p* < 0.05). In conclusion, although it has a different macronutrient composition, the Mediterranean diet may positively affect biochemical parameters and FLI in individuals with NAFLD, albeit in different ways.

## 1. Introduction

Non-alcoholic fatty liver disease (NAFLD) is a broad-spectrum disease that includes steatosis resulting from excessive fat accumulation in the liver, steatohepatitis including ballooning and inflammation in hepatocytes, cirrhosis developing with steatohepatitis, and hepatocellular carcinoma [1]. The American Association for the Study of Liver Diseases (AASL) report stated that NAFLD is observed in 51% of individuals with obesity (body mass index [BMI] > 30 kg/m^2^) [2] and in approximately 75% of individuals with type 2 diabetes [3]. The “double-hit hypothesis”, first proposed by Day et al. in 1998 [4], has been replaced by the “multiple-hit hypothesis”, which involves multiple interlocking processes, including genetic predisposition, environmental factors, dietary habits, insulin resistance, lipotoxicity, oxidative stress, mitochondrial dysfunction and/or endoplasmic reticulum (ER) stress, and changes in the microbiota [5,6]. The diagnosis of NAFLD can be made using a multifaceted clinical evaluation, including a careful anamnesis, physical examination, laboratory findings, liver biopsy, and the most commonly used ultrasonography [7]. However, NAFLD is now also defined as metabolic dysfunction-associated fatty liver disease (MAFLD). Until now, the diagnosis of NAFLD required the exclusion of other chronic liver diseases, including “excessive” alcohol intake. As the pathogenic process leading to MAFLD is now better understood and appears to be caused by an underlying state of systemic metabolic dysfunction, MAFLD is perceived as an independent disease requiring a positive diagnosis rather than a “no disease” definition. Therefore, it was felt that this disease should be defined by its own positive criteria rather than by exclusion criteria. The proposed criteria for a positive diagnosis of MAFLD are histological, imaging, or blood biomarker evidence of hepatosteatosis, in addition to one of the following three criteria: obesity, presence of type 2 diabetes mellitus, or evidence of metabolic dysregulation [8]. However, the term and criteria for NAFLD are used in this article.

The European Association for the Study of the Liver (EASL) reported that the fatty liver index (FLI) is one of the best-validated steatosis scores. The FLI is associated with insulin resistance and reliably predicts the presence but not the severity of steatosis [1]. The FLI has 61% sensitivity and 86% specificity in identifying the presence of steatosis [9], whereas it has 79.8% sensitivity and 71.5% specificity in excluding steatosis at a cut-off point score of <30 [10]. The fibrosis-4 (FIB-4) index is a clinically useful and simple index used for identifying patients who are more likely to have cirrhosis (stage 4) [11]. In the FIB-4 score, a cut-off point of 1.3 has 85% sensitivity and 65% specificity in excluding advanced fibrosis [10].

The high prevalence and complications of NAFLD underline the critical need for a safe, effective, and widely applicable treatment [12]. Lifestyle modification comprising a healthy diet, exercise, and body weight loss is reported as the best approach in the EASL, Italian Association for the Study of the Liver (AISF), Spanish Association for the Study of the Liver, and Asia Pacific Study Group guidelines; although the macronutrient content is different, the Mediterranean diet model is mostly recommended [1,11,13,14,15]. The Mediterranean diet, recognized as an Intangible Cultural Heritage of Humanity by the United Nations Educational, Scientific and Cultural Organization, was proposed and developed by Keys in the 1950s, referring to dietary habits observed in Mediterranean regions [16]. The traditional Mediterranean diet is reported to include high amounts of olive oil, legumes, cereals, vegetables, fruits, and nuts, moderate amounts of dairy products such as cheese and yogurt, fish, white meat, eggs, and wine, and finally, low amounts of meat and meat products [17]. In the Mediterranean diet, fat constitutes 35–45% of the total energy intake, at least half of which should be from monounsaturated fatty acids (MUFAs). Carbohydrates constitute 35–40% and protein 15–20% of the energy intake [18]. MUFA decreases blood triglycerides by increasing fatty acid oxidation by activation of peroxisome proliferator-activated receptor (PPAR)α or by reducing the activation of sterol regulatory element binding protein and inhibiting lipogenesis. The consumption of olive oil showed a significant inverse association with tumor necrosis factor-α and vascular cell adhesion molecule-1 serum levels and improved glycemic tolerance by increased secretion of glucagon-like peptide-1 [19]. There are several mechanisms of action for the effect of eicosapentaenoic acid and docosahexaenoic acid on liver fat content: They affect PPARα and thus promote fatty acid oxidation in the liver; they suppress the expression of two lipogenic transcription factors, sterol regulatory element binding protein 1c and carbohydrate responsive element binding protein, which are involved in de novo lipogenesis of carbohydrates in hyperinsulinemic states; and their increased incorporation into adipose tissue mediates adiponectin production and reduces the susceptibility of adipocytes to high inflammation [20]. For these reasons, the Mediterranean diet can reduce liver steatosis even without a decrease in body weight [21].

In addition to studies showing positive effects on steatosis, inflammation, and fibrosis, studies on the prevention of diabetes and cardiovascular diseases observed with NAFLD also exist [22,23,24].

This study aimed to investigate the effects of TMD, LCMD, and LFMD on biochemical findings, FLI, anthropometric measurements, and body composition in individuals with obesity with NAFLD and insulin resistance.

## 2. Materials and Methods

### 2.1. Study Design and Participants

This study was conducted on 63 participants aged between 18 and 65 years who were insulin resistant and obese (BMI ≥ 30 kg/m^2^), diagnosed with NAFLD by ultrasonography, and admitted to the Department of Internal Medicine and General Surgery at Manavgat State Hospital between June 2021 and September 2022. Individuals who drink alcohol meet the exclusion criteria for NAFLD according to the EASL guidelines [1]; moreover, individuals with types 1 or 2 diabetes, kidney disease, inflammatory bowel disease, cancer, and thyroid dysfunction; are pregnant or breastfeeding; have undergone any surgery in the last 3 months; have taken omega-3, probiotic, vitamin D, and vitamin E supplements in the last 3 months; or are taking insulin or antibiotics were excluded from this study. There are studies showing that probiotics, omega-3, and vitamins D and E have a positive effect on hepatic steatosis and liver enzymes [12,25,26,27,28,29,30,31]. Ethics committee permission was obtained for this experimental study by Acıbadem Mehmet Ali Aydınlar University Medical Research Evaluation Board (ATADEK) (decision number: 2021-09/63). Patients were assigned to the groups by simple random sampling without bias according to the order of admission to the clinical nutrition center, and the dietitian administered “the typical Mediterranean diet (TMD)” to group 1, “the low-carbohydrate Mediterranean diet (LCMD)” to group 2, and “the low-fat Mediterranean diet (LFMD)” to group 3 for 8 weeks. This study spanned a total of 9 weeks, with a pre-interview for each patient.

### 2.2. Dietary Intervention and Monitoring

In the preliminary interview, NAFLD and Mediterranean diets were explained to the patients, and a 14-item questionnaire form was applied to determine the general dietary habits of the individuals. After information about food groups, portions, and weight were explained visually and in writing, the food frequency questionnaire was distributed [32], and the Mediterranean Diet Adherence Scale (MEDAS) form was administered [33]. For the other interview, participants were asked to write their food consumption records for 3 days, including 2 days on weekdays (not consecutive days) and 1 day on weekends, on the food consumption record form [32] with the amounts and bring them in week 1 when the nutritional treatment would start. After anthropometric measurements and body analysis of the participants, total energy requirements were calculated using the Mifflin–St. Jeor equation [34,35], and physical activity coefficient and then dietary interventions with the following macronutrient distribution were applied to the participants by reducing 500 calories of energy. For 8 weeks, feedback was received from the participants who were interviewed face-to-face at the clinical nutrition center every week, and adjustments were made to their diets according to the groups they were in. Food consumption records were taken at the beginning, week 4, and the end of the study.

TMD: 40–45% carbohydrate, 15–20% protein, and 35–40% fat;
LCMD: ≤35% carbohydrate, 15–20% protein, and >45% fat;
LFMD: ≥55% carbohydrate, 15–20% protein, and 20–25% fat.

In all diet types, attention was paid to ensure that the energy intake from saturated fat was <10%, and care was taken to consume at least three portions of vegetables and 2–4 portions of fruits per day and fish twice a week [36]. The meal pattern of the patients was determined as 3 main meals and 2–3 snacks. The obtained food consumption frequencies and food consumption records were analyzed using the BEBIS version 9 program (Nutrition Information System, Istanbul, Türkiye) [37].

### 2.3. Anthropometric and General Characteristics

In this study, participants’ body weight, body fat mass and percentage, abdominal fat mass, visceral fat, and BMI were analyzed using InBody 270 bioelectrical impedance analysis for 9 weeks. Waist and hip circumferences were measured, and the waist/hip circumference ratio was calculated. For waist circumference measurement, the tape was placed midway between the iliac crest and the costal margin of the lower rib and kept horizontal. Participants were asked to look forward and exhale, and the measurement was taken at the end of expiration. For hip circumference measurement, the tape was placed at the widest part of the participant’s hip and below the iliac crest.

In the 23-item questionnaire form for determining the general characteristics of the participants, demographic characteristics, health information, probiotic use, vitamin–mineral supplementation, alcohol and cigarette use, previous dietary treatment, and the symptoms and grade of NAFLD as measured using ultrasonography were recorded.

### 2.4. Physical Activity Assessment

Participants were asked to record their physical activities in minutes within 24 h in the pre-interview and bring them in week 1 when nutritional treatment would be started, and their physical activity levels were evaluated [32]. It was emphasized that they should not change their physical activity during the 8-week study.

### 2.5. Diagnosis, Biochemical Parameters, and Calculated Indexes

Participants referred to the radiology clinic for ultrasonography at the start of the study were diagnosed as having mild adiposity (grade 1), moderate adiposity (grade 2), and severe adiposity (grade 3) according to the ultrasonographic appearance of fatty infiltration. Blood samples collected twice from the participants, at the start of the study and at the end of the eighth week, were examined in the Biochemistry Laboratory of Manavgat State Hospital; the standards and reference values in this laboratory were taken as the basis. In the morning blood samples of the participants after an overnight fast (10–12 h) following dinner, liver enzyme (alanine transaminase [ALT], aspartate aminotransferase [AST], and gamma-glutamyl transferase [GGT]), creatine kinase, total bilirubin, direct bilirubin, hepatitis serology (hepatitis B surface antigen, anti-HBs, and anti-HCV), glycated hemoglobin, fasting blood glucose, fasting insulin, insulin resistance (HOMA-IR), triglyceride, total cholesterol, low-density lipoprotein (LDL), high-density lipoprotein (HDL), triiodothyronine (T3), thyroxine (T4), thyroid-stimulating hormone, and C reactive protein levels were measured, and total cholesterol/HDL cholesterol ratios were calculated [1].

FLI and FIB-4 scores, which are used for differentiating steatosis (Table 1) and advanced fibrosis using available serum parameters, were calculated [38].

The FLI was calculated as follows: (e^(0.953×ln(TG)+0.139×BMI+0.718×ln(GGT)+0.053×WC−15.745)^)/(1+ e^(0.953×ln(TG)+0.139×BMI+0.718×ln(GGT)+0.053×WC−15.745)^) × 100 [39].

**FIB-4 score**: (age × AST)/platelet value × (ALT)^1/2^ [41].
FIB-4 score ≤ 1.3: advanced fibrosis is unlikely.
FIB-4 score > 1.3–<2.67: uncertain.
FIB-4 score ≥ 2.67: patients probably have advanced fibrosis [11,41,42].

### 2.6. Statistical Analyses

In the study, considering two-time variables and three groups, type I error probability α = 0.05, effect size medium effect (0.35), and a targeted power of the test 1 − β = 0.80, the sample size required for statistical analyses was calculated as a total of 63 participants for the three groups, with 21 participants for each group.

All data were recorded and analyzed using Statistical Package for Social Sciences (SPSS version 22.0, IBM, Armonk, NY, USA). To determine the normality of the distribution, the Shapiro–Wilk test, kurtosis and skewness values, which are other assumptions of a normal distribution, and histogram graphs were used. An independent sample *t*-test was used to compare two independent groups, and a paired sample *t*-test was used to analyze the difference between two related numerical variables. One-way analysis of variance was used to compare more than two independent groups, and the Tukey test, one of the multiple comparison tests, was used to determine the source of the difference. Chi-square and Fisher’s exact tests were applied for the relationship between categorical independent variables, and McNemar’s test was applied for the relationship between dependent categorical variables. To examine the relationship between numerical variables, the Pearson correlation coefficient was used. The significance of the findings was evaluated at the *p* < 0.05 level.

## 3. Results

The 8-week medical nutrition intervention was completed with a total of 63 participants. Demographic characteristics, NAFLD grade, physical activity, and MEDAS showed similar distributions in the groups (Table 2).

### 3.1. Participant Food Intake

The evaluation of the changes in dietary macronutrient intake of the groups at the start and end of the study is presented in Table 3. At the end of the study, energy, carbohydrate (g), sucrose (g), protein (g), total fat (g), saturated fatty acid (g), percentage of energy from saturated fat (%), and cholesterol (g) decreased in all three diet groups (*p* < 0.05). As expected, the percentage of energy from carbohydrates, total fat, and fatty acids showed a statistically significant difference between the groups (*p* < 0.05).

### 3.2. Participants’ Anthropometric Measurements

No statistically significant difference was observed between the groups in terms of body composition and anthropometric measurements at the start and end of the study (*p* > 0.05). At the end of the study, body weight, BMI, waist circumference, hip circumference, waist/hip circumference ratio, body fat mass and percentage, abdominal fat mass, and visceral fat were significantly reduced in all three diet groups (*p* < 0.05) (Table 4).

### 3.3. Participants’ Biochemical Parameters and Indexes

At the end of the study, the levels of fasting blood glucose, insulin, insulin resistance, liver enzymes, LDL cholesterol, total cholesterol, total cholesterol/HDL cholesterol ratio, FLI, and FIB-4 scores significantly decreased in the three diet groups (*p* < 0.05), whereas HDL cholesterol levels increased (*p* < 0.05). The TMD group had significantly lower mean AST and GGT values than the other two groups (*p* < 0.05). Moreover, the TMD group had a lower mean FLI score than the LCMD group (*p* < 0.05) and a lower mean FIB-4 score than the LFMD group (*p* < 0.05) (Table 5).

### 3.4. Effects of Macronutrient Changes on the Alterations in Biochemical Parameters

In the TMD group, a positive correlation was noted between the change in the amount of sucrose consumed and the change in the FLI score (r = 0.65, *p* = 0.001) and between the change in the amount of total fat (g) and the changes in ALT (r = 0.56, *p* = 0.007), AST (r = 0.46, *p* = 0.03), and GGT values (r = 0.47, *p* = 0.03) (Table 6).

In the LCMD group, a negative correlation was observed between the change in carbohydrate (g) consumed and the change in the FLI score (r = −0.52, *p* = 0.01), and a positive and moderately significant correlation was noted between the change in sucrose (g) and the changes in ALT (r = 0.49, *p* = 0.02) and AST (r = 0.47, *p* = 0.01). A positive correlation was noted between the changes in protein amount (g) and ALT (r = 0.45, *p* = 0.04), and a negative correlation was observed between the change in the FLI score (r = −0.59, *p* = 0.005). A positive correlation was noted between the change in energy intake from monounsaturated fatty acids and the change in the FLI score (r = 0.44, *p* = 0.04), and a positive correlation was noted between the change in cholesterol and the change in ALT values (r = 0.51, *p* = 0.01).

The change in the amount of fiber consumed was significantly positively correlated with the change in the FLI score (r = 0.45, *p* = 0.03) in the LFMD group. A positive correlation was observed between the change in sucrose and the changes in AST and GGT values (r = 0.43, *p* = 0.04), and a positive correlation was noted between the change in total fat and the change in the FLI score (r = 0.45, *p* = 0.03). The change in energy intake from monounsaturated fatty acids was positively correlated with the changes in ALT (r = 0.45, *p* = 0.03) and GGT values (r = 0.43, *p* = 0.04), whereas a similar relationship was observed with the change in oleic acid values.

The change in the FLI score (r = 0.46, *p* = 0.03) was positively associated with the change in energy intake from polyunsaturated fatty acids. A positive correlation was noted between the change in the FLI score (r = 0.66, *p* = 0.001) (r = 0.64, *p* = 0.002) and the changes in energy intake from saturated fatty acids and cholesterol intake.

## 4. Discussion

Factors such as metabolic diseases, genetics, environmental factors, sex, age, and marital status are significant risk factors for NAFLD [43]. The participants of this study were similar in terms of their general characteristics and NAFLD grades in all groups, which positively contributed to the analyses conducted in this study. The Mediterranean diet is recognized worldwide as one of the healthiest dietary patterns because it reduces the risk of cardiovascular diseases, type 2 diabetes, neurodegenerative diseases, cancer incidence, and overall mortality [44]. It represents an effective dietary approach in NAFLD management as it reduces hepatic steatosis and improves elevated liver transaminase levels. The EASL–EASD–EASO Clinical Practice Guidelines published in 2016 [1] have recommended adjusting the macronutrient composition for medical nutrition therapy according to the Mediterranean diet. However, additional large randomized controlled trials designed to determine the mechanisms underlying the observed effects are needed, and clarification of the exact dietary pattern associated with the beneficial effects of the Mediterranean diet on NAFLD has been reported [45]. The Asia Pacific Research Group, EASL, and AASL have emphasized that individuals with NAFLD should achieve body weight loss by restricting their daily energy intake by 500–1000 calories, whereas AISF has emphasized that they should achieve body weight loss by taking low energy intake, such as 1200–1600 calories/day [13]. In this study, in all three diet groups, individual nutrition plans were formulated by reducing 500 calories from the energy requirements. In this study, the macronutrient consumption of all three groups was within the expected range. In the diet types recommended for NAFLD treatment, adequate intake of whole grain products containing high fiber, fruits, vegetables, legumes, and dried nuts is recommended [46].

Although no exact amount of fiber was mentioned, the American Diabetes Association’s recommendation of 14 g of fiber for every 1000 kcal of daily dietary intake for adults [47] was considered for glycemic control and an improved lipid profile. A study using data from the 2007–2014 National Health and Nutrition Examination Survey revealed that individuals without NAFLD diagnosis received an average of 216 mg/kg of fiber per day, whereas individuals with NAFLD received 156 mg/kg (*p* < 0.001) [48]. At the start of this study, the amount of fiber intake of participants seemed adequate; however, it was not at the desired level according to the energy intake. At the end of the intervention, the amount of fiber intake increased and reached optimal levels. At the end of the study, the LFMD group had significantly higher total fiber, soluble fiber, and insoluble fiber values than the other two groups (*p* < 0.05). Excessive saturated fatty acid intake negatively affected several steps, from insulin resistance to oxidative stress and mitochondrial dysfunction, from hepatic ER stress to increased inflammation in the pathogenesis of NAFLD [49]. Therefore, for NAFLD prevention and treatment, energy intake from saturated fats is recommended to be <10%, which is consistent with the guidelines and the results of previous studies [31,50]. Owing to their positive effects on lipid profile, blood pressure, insulin sensitivity, and glycemic control, monounsaturated fatty acids are the most suitable alternative to saturated fats. Monounsaturated fatty acids reduce the incidence of risk factors associated with metabolic syndrome, especially when consumed as part of the Mediterranean diet [46]. The Spanish Association for the Study of the Liver has reported that in the presence of steatohepatitis, dietary saturated fatty acid intake should be reduced, and n-3 polyunsaturated fatty acid and monounsaturated fatty acid intake should be increased [15]. In the Mediterranean diet, monounsaturated fatty acids should constitute at least 50% of the daily energy intake from fat [18]. Although the intake of monounsaturated fatty acids, including oleic acid (18:1), increases triglyceride content, it reduces cell stress and hepatocellular death [15]. At the end of this study (Table 2), the percentage of saturated fat intake of all participants was below 10%, as targeted, and monounsaturated fatty acids accounted for 50% of their total fat intake. This result shows that participants in all three groups increased their consumption of olive oil, which is at the center of the Mediterranean diet while decreasing other fat sources. In a study conducted by Coppell et al. to examine the relationship between obesity and the extent of liver damage in NAFLD, ALT and GGT levels significantly increased with increasing BMI [51]. In addition, another study showed a significant relationship between waist circumference and waist/hip circumference ratio and NAFLD [52]. Increased visceral fat and insulin resistance that develop in parallel with this increase are the two main actors that play a role in the pathogenesis of NAFLD [53]. In a study, individuals with NAFLD had significantly higher total adipose tissue, subcutaneous adipose tissue, and visceral adipose tissue than those in the control group, and visceral adipose tissue area was independently associated with significant fibrosis (F2–F4) in the multivariate regression analysis [54]. A study conducted by Ristic-Medic et al. observed that a calorie-restricted Mediterranean diet administered to individuals with NAFLD for 3 months significantly reduced BMI (from 30.43 ± 1.81 to 27.65 ± 1.80 kg/m^2^), waist circumference (from 105.67 ± 5.94 to 95.83 ± 5.73 cm), and body fat percentage (from 26.17% ± 1.71% to 21.27% ± 3.05%) [55]. At the end of this study, although significant (*p* < 0.05) changes were observed in terms of body composition and anthropometric measurements in all three diet groups, no significant difference was noted between the groups (*p* > 0.05). Owing to its high fiber content, the Mediterranean dietary pattern increases fermentation and short-chain fatty acid production, thereby promoting reduced insulin resistance. Furthermore, the inclusion of foods with a low glycemic index (LGI), the presence of polyphenols and monounsaturated fatty acids in olive oil, and the increase in omega-3 consumption have curative effects on hyperinsulinemia [56]. At the end of the ATTICA prospective cohort study, which spanned 10 years and included 3042 individuals, the Mediterranean diet adherence score was significantly negatively associated with diabetes risk [57]. In a study in which individuals with NAFLD were administered a calorie-restricted Mediterranean diet and a calorie-restricted low-fat diet, no significant difference was noted between the groups in terms of mean fasting glucose, fasting insulin, and insulin resistance levels [55]. Consistent with previous studies, fasting glucose and insulin resistance significantly decreased in all three diet groups at the end of the study (*p* < 0.05); however, no significant difference was observed between the groups. An energy-restricted Mediterranean diet was administered to individuals with NAFLD, and at the end of the study, individuals with 50% or more compliance with the Mediterranean diet had significantly lower mean ALT, AST, and GGT levels than those with <50% [58]. In the study examining the effect of different diet types on ALT levels in individuals with obesity with type 2 diabetes, the first, second, and third groups were administered the American Diabetes Association diet, LGI diet, and modified Mediterranean diet (MMD), respectively. Although the mean ALT level decreased in all groups at the end of the study, the lowest ALT levels were significantly observed in the MMD group [59]. A 2021 systematic review and meta-analysis emphasized that without calorie restriction, the Mediterranean diet significantly reduced intrahepatic lipid content; however, it did not produce significant reductions in ALT, AST, and GGT values [60]. In contrast, in another systematic review and meta-analysis of randomized controlled trials published in the same year, the Mediterranean diet significantly decreased AST and GGT values; however, it had no significant effect on ALT values. However, with the exclusion of some studies within the scope of the research without publication bias, the Mediterranean diet improved all liver enzymes, and more randomized controlled studies were needed [61]. At the end of this study, mean ALT, AST, and GGT values significantly decreased in all three diet groups (*p* < 0.05) (Table 5). The AST and GGT endpoints of the TMD group were significantly lower than those of the other two groups (*p* < 0.05). As no significant difference was observed between the mean body weight loss of the groups, it is believed that the significantly lower mean AST and GGT values of individuals on the typical Mediterranean diet compared with those in the other two groups may be caused by the difference in macronutrients. The FLI is a noninvasive biomarker recommended before liver ultrasonography to confirm steatosis in individuals with suspected NAFLD. Besides providing convenience in community screening, the FLI has started to be used for the detection of atherosclerosis, diabetes, and chronic kidney diseases [62]. A meta-analysis of systematic reviews published in 2022 reported that the FLI scores of individuals following a calorie-restricted Mediterranean diet protocol were significantly reduced [63]. In the study in which the Mediterranean diet and low-fat diet interventions were applied, FLI scores significantly decreased in both groups; however, the decrease in the Mediterranean diet was significantly higher than that in the other group [55]. In a 10-year prospective cohort study, patients were examined in three groups: low adherence (<25 points), moderate adherence (25–35 points), and high adherence (>35 points) to the Mediterranean diet score, and it was observed that FLI and FIB-4 scores significantly decreased with increasing adherence to the Mediterranean diet [64]. Another study observed that the fibrosis grade significantly decreased with an increasing Mediterranean diet compliance score [65]. In this study, FLI and FIB-4 scores significantly decreased in all three diet groups (*p* < 0.05). The TMD group had a lower mean FLI score (*p* < 0.05) than the LCMD group and a lower mean FIB-4 score (*p* < 0.05) than the LFMD group. It is believed that the reason for these differences is that the biochemical parameters of the participants in the TMD group improved more than those of the other groups.

## 5. Conclusions

The results of this study suggested that the Mediterranean diet can positively affect NAFLD, regardless of macronutrient differences. For example, although the decrease in sucrose and total fat levels in the TMD group positively affected the FLI score and liver enzyme levels, the decrease in sucrose and increase in monounsaturated fatty acid and oleic acid levels in the LCMD group positively affected the FLI score and liver enzyme levels. In the LFMD group, increased intake of fiber, monounsaturated fatty acids, and oleic acids and decreased intake of sucrose, polyunsaturated fatty acids, saturated fatty acids, and cholesterol positively affected the FLI score and liver enzyme levels.

To better understand the effects of Mediterranean diet types containing different macronutrients on biochemical parameters and FLI in NAFLD treatment, randomized controlled trials with longer intervention periods and larger sample sizes are needed.

### 5.1. Strengths and Limitations of the Study

In the literature, no intervention studies have investigated the effectiveness of the Mediterranean diet containing different macronutrients on NAFLD with three different macronutrient patterns. Therefore, this study is the first of its kind.

The strengths of the study can be shown as the preparation of diet lists by meeting face-to-face with all individuals participating in the study every week in the clinical nutrition center of the researcher, taking their body composition and 3-day food consumption records, and evaluating their dietary compliance. The fact that the participants consumed more olive oil and butter at the beginning of the study owing to the characteristics of their geographical region and nutritional culture may have prevented the correlations that would arise from the difference in monounsaturated fatty acid intake during the intervention period from clearly emerging. The limitations of this study include the inability to determine the NAFLD grade by ultrasonography at the end of the study and the short intervention period owing to the coronavirus disease 2019 pandemic.

### 5.2. Importance and Contribution of the Study

In all three diet groups, different biochemical values varied favorably in relation to different macronutrients. This result confirmed the hypothesis of the study: TMD, LCMD, and LFMD positively affect the biochemical findings and FLI of individuals with NAFLD, and a difference exists between these positive effects.

## Figures and Tables

**Table 1:** Fatty liver index chart [39,40].

FLI	Risk	Diagnosis
<30	Low	Fatty liver is excluded
30–<60	Uncertain	Fatty liver can neither be ignored nor excluded
≥60	High	Fatty liver

**Table 2:** Distribution of general characteristics of participants by groups at the start of the study.

	TMD (*n* = 21)		LCMD (*n* = 21)		LFMD (*n* = 21)		
	*n*	%	*n*	%	*n*	%	*p*
Sex							
Females	14	66.67	13	61.90	11	52.38	0.82
Males	7	33.33	8	38.10	10	47.62	
Marital status							
Married	14	66.67	17	80.95	12	57.14	0.31
Single	7	33.33	4	19.05	9	42.86	
Age (years)							
$\bar{X}$ ± SD	39.48 ± 9.17		39.71 ± 10.34		38.62 ± 9.39		0.43
NAFLD grade							
Grade 1	14	66.67	14	66.67	14	66.67	1.00
Grade 2	7	33.33	7	33.33	7	33.33	
Physical activity duration (min/week)							
$\bar{X}$ ± SD	168.13 ± 17.72		171.25 ± 44.22		163.33 ± 20.46		0.96
PAL							
$\bar{X}$ ± SD	1.38 ± 0.05		1.41 ± 0.07		1.38 ± 0.05		0.18
F = 1.75							
MEDAS score							
$\bar{X}$ ± SD	5.52 ± 1.81		5.48 ± 1.75		4.52 ± 1.36		0.83
F = 0.19							

$\bar{X}$, mean; SD, standard deviation; F, one-way analysis of variance; MEDAS, Mediterranean Diet Adherence Scale; LCMD group, low-carbohydrate Mediterranean diet group; LFMD group, low-fat Mediterranean diet group; TMD group, typical Mediterranean diet group; PAL, physical activity level; *p*, baseline differences between groups.

**Table 3:** Evaluation of the changes in macronutrients of participants at the start and end of the study by groups.

Macronutrient	Intervention Status	^1^ TMD (*n* = 21) $\bar{X}$ ± SD	^2^ LCMD (*n* = 21) $\bar{X}$ ± SD	^3^ LFMD (*n* = 21) $\bar{X}$ ± SD	Between-Group Comparison $\bar{X}$ ± SD	Difference
Energy (kcal)	Pre-intervention	3248.45 ± 459.96	3209.79 ± 525.43	3366.13 ± 575.26	F = 0.51; *p* = 0.60	
	Post-intervention	1718.68 ± 260.12	1803.20 ± 324.59	1785.72 ± 319.72	F = 0.46; *p* = 0.64	
	Intra-group comparison	t = 19.25; *p* = 0.00	t = 19.00; *p* = 0.00	t = 17.28; *p* = 0.00		
Carbohydrate (g)	Pre-intervention	345.78 ± 58.20	358.31 ± 62.54	362.89 ± 53.26	F = 0.49; *p* = 0.62	
	Post-intervention	174.95 ± 25.70	148.54 ± 27.01	241.81 ± 42.73	F = 45.28; *p* = 0.00	2 < 1, 3 and 1 < 3
	Intra-group comparison	t = 13.94; *p* = 0.00	t = 20.54; *p* = 0.00	t = 12.08; *p* = 0.00		
Carbohydrate (%)	Pre-intervention	43.55 ± 4.37	45.67 ± 3.35	44.38 ± 2.95	F = 1.84; *p* = 0.17	
	Post-intervention	42.10 ± 1.14	34.05 ± 0.67	55.71 ± 0.56	F = 3681.93; *p* = 0.00	1, 2 < 3 and 2 < 1
	Intra-group comparison	t = 1.44; *p* = 0.17	t = 14.37; *p* = 0.00	t = −18.62; *p* = 0.00		
Fiber (gr)	Pre-intervention	31.25 ± 6.07	30.37 ± 5.96	30.49 ± 6.69	F = 0.12; *p* = 0.88	
	Post-intervention	37.89 ± 4.92	38.30 ± 6.13	56.15 ± 14.41	F = 25.42; *p* = 0.00	1, 2 < 3
	Intra-group comparison	t = −3.47; *p* = 0.00	t = −4.46; *p* = 0.00	t = −7.57; *p* = 0.00		
Sucrose (g)	Pre-intervention	77.90 ± 27.55	84.07 ± 28.49	78.98 ± 24.77	F = 0.31; *p* = 0.73	
	Post-intervention	26.96 ± 7.35	24.59 ± 6.37	28.51 ± 8,20	F = 1.52; *p* = 0.23	
	Intra-group comparison	t = 8.66; *p* = 0.00	t = 9.68; *p* = 0.00	t = 9.26; *p* = 0.00		
Protein (g)	Pre-intervention	116.67 ± 20.22	117.86 ± 21.01	123.14 ± 23.15	F = 0.54; *p* = 0.59	
	Post-intervention	80.08 ± 13.02	81.51 ± 14.55	83.84 ± 16.04	F = 0.36; *p* = 0.70	
	Intra-group comparison	t = 10.25; *p* = 0.00	t = 13.14; *p* = 0.00	t = 8.81; *p* = 0.00		
Protein (%)	Pre-intervention	14.74 ± 1.95	15.21 ± 1.79	14.93 ± 1.32	F = 0.41; *p* = 0.66	
	Post-intervention	19.14 ± 1.01	18.71 ± 0.85	19.33 ± 0.66	F = 2.91; *p* = 0.06	
	Intra-group comparison	t = −8.59; *p* = 0.00	t = −8.42; *p* = 0.00	t = −12.73; *p* = 0.00		
Total fat (g)	Pre-intervention	152.28 ± 27.06	142.11 ± 29.78	154.88 ± 34.40	F = 1.02; *p* = 0.37	
	Post-intervention	73.94 ± 11.56	94.37 ± 17.43	49.78 ± 9.21	F = 60.06; *p* = 0.00	1, 3 < 2 and 3 < 1
	Intra-group comparison	t = 16.20; *p* = 0.00	t = 8.52; *p* = 0.00	t = 17.21; *p* = 0.00		
Total fat (%)	Pre-intervention	41.57 ± 3,84	39.21 ± 3.64	40.74 ± 3,02	F = 2.43; *p* = 0.10	
	Post-intervention	38.71 ± 1.06	47.14 ± 0.91	24.90 ± 0.30	F = 3905.36; *p* = 0.00	1, 3 < 2 and 3 < 1
	Intra-group comparison	t = 3.11; *p* = 0.01	t = −8.60; *p* = 0.00	t = 24.45; *p* = 0.00		
Saturated fatty acid (%)	Pre-intervention	16.57 ± 2.25	16.15 ± 2.25	16.55 ± 2.52	F = 0.22; *p* = 0.81	
	Post-intervention	7.93 ± 0.49	8.85 ± 0.47	4.29 ± 0.45	F = 545.73; *p* = 0.00	1, 3 < 2 and 3 < 1
	Intra-group comparison	t = 18.22; *p* = 0.00	t = 14.15; *p* = 0.00	t = 21.73; *p* = 0.00		
Monounsaturated fatty acid (%)	Pre-intervention	16.20 ± 2.11	14.85 ± 1.98	15.02 ± 2.18	F = 2.57; *p =* 0.09	
	Post-intervention	19.46 ± 1.25	25.88 ± 0.83	14.77 ± 0.53	F = 773.02; *p =* 0.00	1, 3 < 2 and 3 < 1
	Intra-group comparison	t = −6.94; *p* = 0.00	t = −24.51; *p* = 0.00	t = 0.52; *p* = 0.61		
Oleic acid (%)	Pre-intervention	14.64 ± 1.95	13.25 ± 1.91	13.44 ± 2.07	F = 3.03; *p* = 0.06	
	Post-intervention	18.40 ± 1.43	25.14 ± 0.86	14.35 ± 0.65	F = 582.44; *p* = 0.00	1, 3 < 2 and 3 < 1
	Intra-group comparison	t = −7.46; *p* = 0.00	t = −27.90; *p* = 0.00	t = −1.95; *p* = 0.06		
Polyunsaturated fatty acid (%)	Pre-intervention	6.40 ± 1.84	5.84 ± 1.41	6.71 ± 2.16	F = 1.22; *p* = 0.30	
	Post-intervention	9.06 ± 0.78	9.51 ± 0.98	4.32 ± 0.57	F = 274.56; *p* = 0.00	3 < 1, 2
	Intra-group comparison	t = −7.74; *p* = 0.00	t = −9.31; *p* = 0.00	t = 4,85; *p* = 0.00		
Cholesterol (mg)	Pre-intervention	609.25 ± 164.18	530.22 ± 115.77	601.34 ± 186.70	F = 1.59; *p* = 0.21	
	Post-intervention	93.93 ± 22.44	101.22 ± 23.81	67.60 ± 18.76	F = 13.85; *p* = 0.00	1, 3 < 2 and 3 < 1
	Intra-group comparison	t = 14.89; *p* = 0.00	t = 16.90; *p* = 0.00	t = 13.22; *p* = 0.00		

$\bar{X}$, mean; SD, standard deviation; F, one-way analysis of variance; t, paired sample *t*-test; ^1^, typical Mediterranean diet group; ^2^, low-carbohydrate Mediterranean diet group; ^3^, low-fat Mediterranean diet group.

**Table 4:** Evaluation of body compositions and anthropometric measurements of participants at the start and end of the study.

Body Composition Anthropometric Measurements	Intervention Status	^1^ TMD (*n* = 21) $\bar{X}$ ± SD	^2^ LCMD (*n* = 21) $\bar{X}$ ± SD	^3^ LFMD (*n* = 21) $\bar{X}$ ± SD	Between-Group Comparison $\bar{X}$ ± SD	Difference
Body weight (kg)	Pre-intervention	91.90 ± 10.94	93.71 ± 12.36	94.12 ± 11.44	F = 0.40; *p* = 0.67	
	Post-intervention	84.43 ± 10.34	86.87 ± 12.15	86.71 ± 10.87	F = 0.17; *p* = 0.84	
	Intra-group comparison	t = 23.93; *p* = 0.00	t = 19.02; *p* = 0.00	t = 32.61; *p* = 0.00		
BMI (kg/m^2^)	Pre-intervention	32.30 ± 1.08	32.46 ± 1.68	32.34 ± 1.19	F = 0.22; *p* = 0.81	
	Post-intervention	29.74 ± 1.22	30.08 ± 1.67	29.80 ± 1.23	F = 0.28; *p* = 0.76	
	Intra-group comparison	t = 27.26; *p* = 0.00	t = 17,04; *p* = 0.00	t = 38.39; *p* = 0.00		
Waist circumference (cm)	Pre-intervention	109.93 ± 7.40	114.71 ± 14.78	111.19 ± 6,55	F = 0.31; *p* = 0.73	
	Post-intervention	100.52 ± 7.35	106.10 ± 13.81	102.21 ± 6.81	F = 0.30; *p* = 0.74	
	Intra-group comparison	t = 22.90; *p* = 0.00	t = 14.80; *p* = 0.00	t = 24.21; *p* = 0.00		
Hip circumference (cm)	Pre-intervention	106.71 ± 6.66	113.90 ± 11.36	108.05 ± 8.90	F = 0.08; *p* = 0.92	
	Post-intervention	100.90 ± 6.60	108.10 ± 10.69	102.38 ± 8.89	F = 0.52; *p* = 0.60	
	Intra-group comparison	t = 20.06; *p* = 0.00	t = 17.90; *p* = 0.00	t = 17.68; *p* = 0.00		
Waist/hip circumference	Pre-intervention	1.03 ± 0.07	1.01 ± 0.12	1.03 ± 0.06	F = 0.35; *p* = 0.70	
	Post-intervention	1.00 ± 0.07	0.98 ± 0.13	0.99 ± 0.05	F = 0.36; *p* = 0.70	
	Intra-group comparison	t = 4.35; *p* = 0.00	t = 6.24; *p* = 0.00	t = 6.25; *p* = 0.00		
Body fat percentage (%)	Pre-intervention	40.14 ± 6.14	38.97 ± 6.22	37.99 ± 7.96	F = 1.77; *p* = 0.18	
	Post-intervention	35.52 ± 6.58	35.24 ± 5.94	33.92 ± 8.61	F = 0.69; *p* = 0.50	
	Intra-group comparison	t = 13.64; *p* = 0.00	t = 13.45; *p* = 0.00	t = 17.90; *p* = 0.00		
Abdominal fat mass (kg)	Pre-intervention	18.91 ± 1.84	18.42 ± 2.53	18.10 ± 2.65	F = 0.62; *p* = 0.54	
	Post-intervention	15.30 ± 2.12	14.91 ± 2.37	14.41 ± 2.81	F = 0.69; *p* = 0.50	
	Intra-group comparison	t = 17.69; *p* = 0.00	t = 16.10; *p* = 0.00	t = 20.68; *p* = 0.00		

$\bar{X}$, mean; SD, standard deviation; F, one-way analysis of variance; t, paired sample *t*-test; ^1^, typical Mediterranean diet group; ^2^, low-carbohydrate Mediterranean diet group; ^3^, low-fat Mediterranean diet group.

**Table 5:** Evaluation of the biochemical parameters of participants at the start and end of the study by groups.

Biochemical Parameters	Intervention Status	^1^ TMD (*n* = 21) $\bar{X}$ ± SD	^2^ LCMD (*n* = 21) $\bar{X}$ ± SD	^3^ LFMD (*n* = 21) $\bar{X}$ ± SD	Between-Group Comparison $\bar{X}$ ± SD	**Difference**
Fasting blood glucose (mg/dL)	Pre-intervention	123.29 ± 17.08	117.14 ± 13.33	114.14 ± 13.47	F = 2.10; *p* = 0.13	
	Post-intervention	106.05 ± 13.67	103.05 ± 10.93	102.71 ± 10.79	F = 0.50; *p* = 0.61	
	Intra-group comparison	t = 11.26; *p* = 0.01	t = 9.61; *p* = 0.01	t = 8.38; *p* = 0.01		
HOMA-IR	Pre-intervention	4.24 ± 0.70	3.85 ± 0.70	4.24 ± 1.00	F = 1.60; *p* = 0.21	
	Post-intervention	2.38 ± 0.46	2.50 ± 0.55	2.67 ± 0.80	F = 1.17; *p* = 0.32	
	Intra-group comparison	t = 18.32; *p* = 0.01	t = 15.05; *p* = 0.01	t = 18.38; *p* = 0.01		
ALT (U/L)	Pre-intervention	69.19 ± 14.48	66.38 ± 11.36	68.67 ± 15.30	F = 0.25; *p* = 0.78	
	Post-intervention	48.52 ± 9.00	51.43 ± 6.34	51.95 ± 10.79	F = 0.90; *p* = 0.41	
	Intra-group comparison	t = 11.00; *p* = 0.01	t = 7.60; *p* = 0.01	t = 9.66; *p* = 0.01		
AST (U/L)	Pre-intervention	42.19 ± 13.36	45.71 ± 14.62	50.43 ± 16.50	F = 1.62; *p* = 0.21	
	Post-intervention	26.76 ± 7.08	34.90 ± 8.17	37.24 ± 10.60	F = 8.31; *p* = 0.01	1 < 2, 3
	Intra-group comparison	t = 7.90; *p* = 0.01	t = 6.14; *p* = 0.01	t = 7.29; *p* = 0.01		
GGT (U/L)	Pre-intervention	35.38 ± 7.05	39.33 ± 11.13	43.29 ± 12.10	F = 3.08; *p* = 0.05	1 < 3
	Post-intervention	20.81 ± 5.73	31.24 ± 8.17	30.76 ± 8.26	F = 13.02; *p* = 0.01	1 < 2, 3
	Intra-group comparison	t = 10.81; *p* = 0.01	t = 7.11; *p* = 0.01	t = 9.06; *p* = 0.01		
FLI	Pre-intervention	85.62 ± 7.34	87.90 ± 8.79	88.71 ± 4.77	F = 1.06; *p* = 0.35	
	Post-intervention	60.38 ± 13.15	71.95 ± 17.18	68.71 ± 10.97	F = 3.82; *p* = 0.03	1 < 2
	Intra-group comparison	t = 13.97; *p* = 0.01	t = 7.90; *p* = 0.01	t = 13.53; *p* = 0.01		
FIB-4	Pre-intervention	0.61 ± 0.22	0.73 ± 0.28	0.73 ± 0.22	F = 1.64; *p* = 0.20	
	Post-intervention	0.48 ± 0.16	0.61 ± 0.20	0.62 ± 0.17	F = 4.10; *p* = 0.02	1 < 3
	Intra-group comparison	t = 6.84; *p* = 0.01	t = 4.59; *p* = 0.01	t = 5.53; *p* = 0.01		

$\bar{X}$, mean; SD, standard deviation; F, one-way analysis of variance; t, paired sample *t*-test; ALT, alanine aminotransferase; AST, aspartate aminotransferase; FLI, fatty liver index; FIB-4, fibrosis-4 score; GGT, gamma-glutamyl transferase. ^1^, typical Mediterranean diet group; ^2^, low-carbohydrate Mediterranean diet group; ^3^, low-fat Mediterranean diet group.

**Table 6:** Relationship between changes in liver enzyme values and FLI scores of the groups at the beginning and end of the study, and the change values of macronutrients.

		^1^ TMD (*n* = 21) $\bar{X}$ ± SD				^2^ LCMD (*n* = 21) $\bar{X}$ ± SD				^3^ LFMD (*n* = 21) $\bar{X}$ ± SD			
		ALT	AST	GGT	FLI	ALT	AST	GGT	FLI	ALT	AST	GGT	FLI
Carbohydrate (g)	r	0.41	0.29	0.43	0.38	0.42	0.28	0.16	−0.52	−0.15	−0.34	−0.29	0.24
	*p*	0.06	0.19	0.05	0.89	0.054	0.21	0.47	0.01	0.50	0.12	0.19	0.29
Carbohydrate (%)	r	0.02	−0.49	0.09	0.17	0.21	0.20	0.38	−0.18	0.08	−0.01	−0.07	−0.16
	*p*	0.90	0.83	0.68	0.44	0.35	0.36	0.84	0.42	0.71	0.94	0.73	0.46
Fiber (g)	r	0.22	0.04	0.21	−0.17	0.15	0.12	0.06	−0.03	0.08	0.07	0.08	0.45
	*p*	0.32	0.86	0.34	0.46	0.50	0.57	0.77	0.87	0.70	0.75	0.70	0.03
Sucrose (g)	r	0.24	0.20	0.33	0.65	0.49	0.47	0.26	−0.31	0.12	0.43	0.43	0.28
	*p*	0.28	0.37	0.13	0.001	0.02	0.01	0.24	0.17	0.35	0.04	0.04	0.20
Protein (g)	r	0.15	0.31	0.08	−0.19	0.45	0.23	−0.13	−0.59	−0.14	−0.22	−0.16	0.09
	*p*	0.51	0.16	0.71	0.40	0.04	0.29	0.56	0.005	0.53	0.31	0.48	0.68
Protein (%)	r	−0.31	−0.10	−0.33	−0.40	0.19	0.09	−0.04	−0.35	−0.10	−0.09	−0.12	−0.23
	*p*	0.17	0.66	0.13	0.07	0.39	0.67	0.86	0.11	0.65	0.68	0.59	0.30
Total fat (g)	r	0.56	0.46	0.47	0.27	−0.13	−0.14	−0.25	0.06	−0.10	−0.16	−0.5	0.45
	*p*	0.007	0.03	0.03	0.22	0.54	0.52	0.25	0.77	0.65	0.46	0.80	0.03
Total fat (%)	r	0.16	0.14	0.08	0.01	−0.27	−0.20	−0.30	0.27	−0.05	0.02	0.11	0.24
	*p*	0.47	0.54	0.72	0.94	0.23	0.36	0.18	0.22	0.81	0.90	0.61	0.29
SFA (%)	r	0.32	0.19	0.19	0.28	−0.25	0.14	0.23	0.29	0.26	−0.28	0.24	0.66
	*p*	0.15	0.40	0.39	0.21	0.26	0.53	0.31	0.22	0.25	0.20	0.28	0.001
MUFA (%)	r	0.11	0.18	0.09	0.12	0.26	0.24	0.29	0.44	0.45	0.39	0.43	0.09
	*p*	0.61	0.42	0.69	0.60	0.25	0.28	0.20	0.04	0.03	0.07	0.04	0.66
Oleic acid (%)	r	0.03	0.08	0.01	0.08	0.30	0.28	0.30	0.42	0.50	0.43	0.43	0.09
	*p*	0.87	0.72	0.97	0.71	0.18	0.20	0.17	0.052	0.02	0.05	0.02	0.66
PUFA (%)	r	−0.02	−0.05	−0.02	−0.39	−0.12	−0.11	−0.17	−0.03	−0.15	0.03	0.02	0.46
	*p*	0.91	0.81	0.92	0.07	0.60	0.61	0.45	0.86	0.50	0.89	0.90	0.03
Cholesterol (mg)	r	0.06	0.15	0.14	0.38	0.51	0.15	−0.17	−0.25	0.03	−0.13	−0.08	0.64
	*p*	0.77	0.51	0.95	0.08	0.01	0.50	0.44	0.28	0.87	0.56	0.71	0.002

$\bar{X}$, mean; SD, standard deviation; MUFA, monounsaturated fatty acid; PUFA, polyunsaturated fatty acid; SFA, saturated fatty acid; ^1^, typical Mediterranean diet group; ^2^, low-carbohydrate Mediterranean diet group; ^3^, low-fat Mediterranean diet group.

## Data Availability

The data obtained in this study are available from the corresponding author upon request. The data are not publicly available due to ethical reasons.
